# Discovery Proteomics Analysis Determines That Driver Oncogenes Suppress Antiviral Defense Pathways Through Reduction in Interferon-β Autocrine Stimulation

**DOI:** 10.1016/j.mcpro.2022.100247

**Published:** 2022-05-18

**Authors:** Paige E. Solomon, Lisa L. Kirkemo, Gary M. Wilson, Kevin K. Leung, Mark H. Almond, Leanne C. Sayles, E. Alejandro Sweet-Cordero, Oren S. Rosenberg, Joshua J. Coon, James A. Wells

**Affiliations:** 1 Department of Pharmaceutical Chemistry, University of California, San Francisco, San Francisco, California, USA; 2 Department of Chemistry, University of Wisconsin-Madison, Madison, Wisconsin, USA; 3 Department of Biomolecular Chemistry, University of Wisconsin-Madison, Madison, Wisconsin, USA; 4 Division of Infectious Diseases, Department of Medicine, UCSF Medical Center, University of California, San Francisco, California, USA; 5 Department of Pediatrics, University of California San Francisco, California, USA; 6 Department of Biophysics and Biochemistry, Chan Zuckerberg Biohub, San Francisco, California, USA; 7 National Center for Quantitative Biology of Complex Systems, University of Wisconsin-Madison, Madison, Wisconsin, USA; 8 Department of Cellular and Molecular Pharmacology, University of California, San Francisco, San Francisco, California, USA

**Keywords:** oncogenes, MYC, KRAS, BRAF, MEK, AKT, HER2, EGFR, interferon, double-stranded RNA, AKT, AKT serine/threonine kinase 1, BRAF, serine/threonine-protein kinase B-Raf, cGAMP, cyclic GMP-AMP, dsDNA, double-stranded DNA, dsRNA, double-stranded RNA, EGFR, epidermal growth factor receptor, HER2, human epidermal growth factor receptor-2, IFN, interferon, ISG, interferon-stimulated gene, ISRE, interferon-stimulated response element, KRAS, K-Ras GTPase, LFQ, label-free quantitation, MEK, MEK1 protein kinase, MHC, major histocompatibility complexes, MOI, multiplicity of infection, MYC, c-Myc, OS, osteosarcoma, PDAC, pancreatic ductal adenocarcinoma, PDX, patient-derived xenograft, polyI:C, polyinosinic-polycytidylic acid, T1IFN, Type 1 interferon

## Abstract

Since the discovery of oncogenes, there has been tremendous interest to understand their mechanistic basis and to develop broadly actionable therapeutics. Some of the most frequently activated oncogenes driving diverse cancers are c-MYC, EGFR, HER2, AKT, KRAS, BRAF, and MEK. Using a reductionist approach, we explored how cellular proteomes are remodeled in isogenic cell lines engineered with or without these driver oncogenes. The most striking discovery for all oncogenic models was the systematic downregulation of scores of antiviral proteins regulated by type 1 interferon. These findings extended to cancer cell lines and patient-derived xenograft models of highly refractory pancreatic cancer and osteosarcoma driven by KRAS and MYC oncogenes. The oncogenes reduced basal expression of and autocrine stimulation by type 1 interferon causing remarkable convergence on common phenotypic and functional profiles. In particular, there was dramatically lower expression of dsRNA sensors including DDX58 (RIG-I) and OAS proteins, which resulted in attenuated functional responses when the oncogenic cells were treated with the dsRNA mimetic, polyI:C, and increased susceptibility to infection with an RNA virus shown using SARS-CoV-2. Our reductionist approach provides molecular and functional insights connected to immune evasion hallmarks in cancers and suggests therapeutic opportunities.

## Highlights

• Proteomics for isogenic and cancer-derived models of c-MYC, AKT, BRAF, EGFR, HER2, KRAS, and MEK.
• Bidirectional changes to hundreds of proteins that converged at the pathways level.
• Systematic downregulation of antiviral proteins regulated by type 1 interferon.
• Low expression of dsRNA sensors caused desensitization to RNA ligands and susceptibility to RNA virus.

## Graphical Abstract

**Figure:**
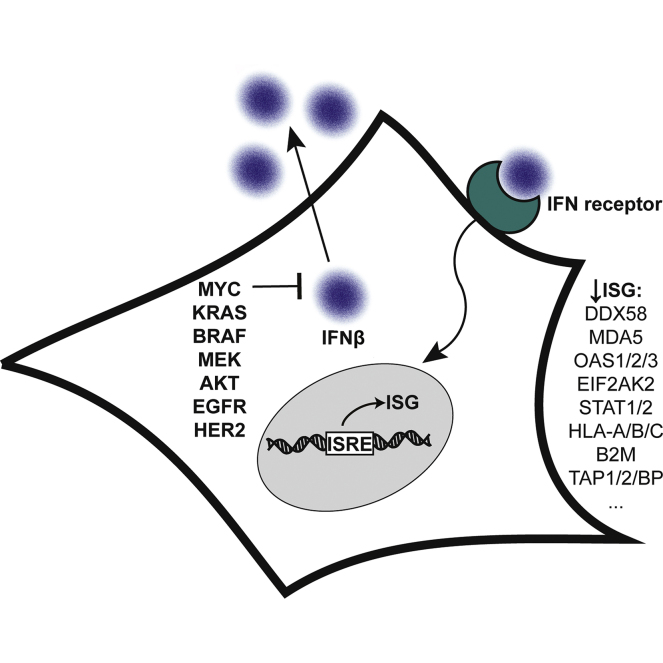

## In Brief

In this work, we systematically evaluated proteome remodeling driven by well-known oncogenes (c-Myc, EGFR, HER2, AKT, KRAS, BRAF, or MEK) expressed in isogenic models and relevant cancer-derived and patient-derived pancreatic cancer and osteosarcoma. We discovered that these driver oncogenes ubiquitously and dramatically suppressed proteins involved in interferon and antiviral defense pathways. Comprehensive functional and molecular analyses showed that cells expressing oncogenes have reduced interferon autocrine stimulation, impaired dsRNA sensing, and increased susceptibility to infection with an RNA virus. Our results reinforce the direct role of driver oncogenes in establishing an immune evasion phenotype and have immediate relevance to the efficacies of therapies that utilize interferon and antiviral pathways.

Cancer is dominated by a set of driver oncogenes that remodel cellular physiology to achieve hallmarks of the disease (1, 2). c-MYC (MYC), epidermal growth factor receptor (EGFR), human epidermal growth factor receptor-2 (HER2), AKT serine/threonine kinase 1 (AKT), K-Ras GTPase (KRAS), serine/threonine-protein kinase B-Raf (BRAF), and MEK1 protein kinase (MEK) are classic examples of powerful oncogenes that activate several distinct tumorigenic axes in cancers (3, 4, 5). For example, MYC is a master transcriptional regulator for thousands of genes that coordinate cellular proliferation and biogenesis (6, 7). MYC is dysregulated in more than 50% of cancers of all tissues but is especially implicated in prostate cancers and B-cell cancers such as Burkitt’s lymphoma, which is driven by chromosomal translocation of MYC (7, 8, 9). MYC copy number amplification has also been correlated to the metastatic progression of osteosarcoma (OS), a predominantly pediatric bone cancer that becomes fatal in advanced disease (10, 11). The signal transduction oncogenes regulate the MAPK (KRAS/BRAF/MEK) or PI3K/AKT proliferation pathways that are activated by the growth receptors EGFR and HER2. Mutant KRAS is the most prominent oncogene in human cancers, and in particular, pancreatic cancer carries the highest rate of mutation to KRAS and is one of the most lethal type of tumor (12, 13, 14). Another signaling oncogene is AKT, a kinase that functions within the PI3K proliferative transduction pathway. Activation of AKT is present in many cancers, including over 40% of breast cancers and is a predictor of poor prognosis and drug resistance (15, 16).

There is considerable interest in understanding the molecular changes induced by driver oncogenes to identify unifying hallmarks and broader drug targets (1, 2). Molecular studies using cancer cell lines, patient-derived xenograft (PDX) models, and primary tumors clearly demonstrate that tumor development drives massive multi-omics changes. A challenge is that these human cancer-derived systems usually have unique combinations of genomic mutations making it difficult to attribute specific molecular changes to each oncogene and confounding the generalizability for target discovery. To reduce the complexity, investigators have used isogenic cell lines that knockout or overexpress specific oncogenes to measure the consequences of isolated molecular perturbations. These reductionist experiments can systematically define the changes driven by oncogenes, building fundamental knowledge to interrogate diverse cancers. Although not based directly on complex primary human tumors, isogenic studies allow control of a single gene and are renewable platforms to identify common hallmarks and broad drug targets across oncogenes.

We previously engineered a series of isogenic cell lines with or without seven different driver oncogenes, MYC, EGFR, HER2, AKT, KRAS, BRAF, and MEK, to specifically identify membrane proteins that change for targeting by immunotherapy (17, 18). Here, we apply discovery proteomics for each of these isogenic cell lines to understand oncogene-driven remodeling of the cytosolic proteome and to identify conserved dysregulation across multiple oncogenes. The most remarkable result for all oncogenes and models tested was the downregulation of type 1 interferon (T1IFN) and antiviral response proteins, especially those associated with viral dsRNA sensing. This effect was also dramatically seen in two PDX models of metastatic OS with high MYC copy number as well as two pancreatic ductal adenocarcinoma (PDAC) cell lines driven by KRAS mutation and MYC amplification. Using systematic molecular and functional analyses, we demonstrate cells expressing oncogenes have impaired dsRNA-sensing antiviral responses and increased susceptibility to RNA virus. These findings are relevant to immune evasion hallmarks in cancer and have implications for the efficacies of radiation, genotoxic, epigenetic, immune, and viral therapies that utilize interferon and antiviral pathways.

## Experimental Procedures

### Generating PDX Cell Lines

PDX tumors were grown in NSG mice. Once large tumors formed, they were resected and minced with a razor blade and digested to make a single-cell suspension using either collagenase digestion buffer or BD tumor dissociation reagent (BD Biosciences Cat# 661563) shaking at 37 °C for 1 h. Cells were filtered through 70 μm mesh and washed twice in Dulbecco's modified Eagle's medium (DMEM)/F12 (Gibco Cat #21331020) supplemented with 10% fetal bovine serum (FBS) (Gibco) and 1% PSG (Gibco Cat# 10378016). Cells were plated in standard tissue culture conditions and allowed to expand. After several weeks, human cells were isolated from mouse stroma by FACS using human HLA-A,B,C antibody (BioLegend Cat# 11414). Cells were allowed to expand for several weeks and sorted a second time to generate a pure population. Cell lines were submitted for STR (IDEXX BioAnalytics) and determined to match the PDX from which they were derived and were confirmed *mycoplasma* free. Cell lines were also submitted for low pass WGS to confirm that they match the patient from which they were derived and PDX.

### Culturing Cell Lines

P493-6 cell lines were cultured in RPMI media (Cytiva, Cat# SH30027.01) with 10% tetracycline-negative FBS (Gemini Bio-Products, Cat# 100–108) and 1% penicillin/streptomycin (Thermo Fisher Scientific, Cat# 15–140–122). MYC expression was repressed in P493-6 cells by treatment with 1 μg/ml tetracycline (Sigma Aldrich, Cat# T7660–25G) for 48 h before downstream analyses. LHS cell lines were cultured in RPMI media with 10% FBS (Gemini Bio-Products, Cat# 100–106) and 1% penicillin/streptomycin. MCF10A cell lines were cultured in DMEM media (Cytiva, Cat# SH30022.01) with 5% horse serum (Gemini Bio-Products, Cat# 999–999 custom sera), 1% penicillin/streptomycin, 20 ng/ml epidermal growth factor (Thermo Fisher Scientific, Cat# PHG0311), 0.5 mg/ml hydrocortisone, 100 ng/ml cholera toxin (Sigma Aldrich, Cat# C8052-2 MG), and 10 μg/ml insulin (Sigma Aldrich, I0516-5 Ml). PDX cell lines and human fetal osteoblasts were culture in DMEM media with 10% bovine growth serum and 1% penicillin/streptomycin. PDAC cell lines were cultured in Isocove Modified Dulbecco media (UCSF Cell Culture Facility) with 10% FBS and 1% penicillin/streptomycin. HPDE-6E6/E7 cells were cultured in Keratinocyte SFM (Thermo Scientific, Cat# 17005042) with 25 mg of bovine pituitary extract and 2.5 μg of epidermal growth factor. All cells were maintained at 37 ^°^C and 5% CO_2_.

### Whole Cell Label-Free Proteomics

#### LHS, P493-6, OS, PDAC, and GSK8612 Treatment

Cell lines were analyzed in biological triplicate. Cell pellets were washed in PBS and resuspended in preheated lysis buffer (filtered 50 mM Tris pH 8.5 containing 6M guanidinium hydrocholoride (GdnHCl) (Chem Impex, Cat# 00152), 5 mM TCEP (MilliporeSigma, Cat# 5805601 GM), and 10 mM chloroacetamide (Sigma Aldrich, Cat# C0267–100G)). Samples were boiled at 97 °C for 10 min with interim mixing. Insoluble debris was removed by centrifugation for 10 min at 21,000*g*, and supernatants were diluted using filtered 50 mM Tris pH 8.5 to achieve a final GdnHCl concentration of 2M. Protein absorbance at 280 nm was measured to determine lysate protein concentrations, and 1 μg of trypsin (Thermo Fisher Scientific, Cat# 90057) per 100 μg of protein was added. After overnight digestion, samples were desalted using C18 columns (Thermo Scientific, Cat# 60109–001 or Thermo Scientific, Cat# 89873). Eluted peptides were lyophilized. Injections of peptides for LC-MS mass spectrometry were prepared by resuspending peptides in 2% acetonitrile (Fisher Scientific, Cat# A955–4) and 0.1% formic acid (Fisher Scientific, Cat# A117–50) solution. 1.5 μg of peptide was injected into an UltiMate 3000 UHPLC system (Thermo Fischer Scientific) with a prepacked Acclaim PepMap C18 reversed phase column (Thermo Fisher Scientific, Cat# DX164534) attached to a Q Exactive Plus mass spectrometer (Thermo Fisher Scientific). Peptides were separated using a linear gradient of 3 to 35% solvent B (Solvent A: 0.1% formic acid, solvent B: 80% acetonitrile, 0.1% formic acid) over 230 min at 300 μl/min. Data-dependent acquisition mode using a top 20 method was utilized for analysis (dynamic exclusion 35 s, selection of peptides charge 2 to 4). Full MS1 spectra were gathered using resolution of 140,000 (at 200 m/z), AGC target of 3e6, maximum injection time of 120 ms, and scan range 400–1800 m/z. MS2 scans were collected at resolution of 17,500 (at 200 m/z) and AGC target of 5e4, maximum injection time of 60 ms, collision energy of 27, and isolation window and offset of 1.5 and 0.5 m/z, respectively. MaxQuant (Version 1.6.7) software was used to analyze chromatograms, to search Uniprot Human Reference Proteome spectral library (downloaded July 2019; 219,758 entries searched in database), and to perform label-free quantitation (LFQ) (19). Peptides were searched using full-tryptic cleavage constraints with maximum two missed or nonspecific cleavages. Searches were performed with precursor mass tolerance of 20 ppm and product ion mass tolerance of 0.5 Da. Cysteine carbamidomethyl was set as a fixed modification; N-terminal acetylation, methionine oxidation, and N-terminal glutamate to pyroglutamate were set as variable modifications. Search results were filtered to a false discovery of 1% at both the peptide and proteins levels. The mass spectrometry proteomics data have been deposited to the ProteomeXchange Consortium *via* the PRIDE partner repository with the dataset identifier PXD033373 (20).

#### MCF10A Proteomics

Cell lines were analyzed in biological triplicate. 20 × 10^6^ cells were suspended in 200 μl 6 M guanidine HCl and boiled for 5 min at 100 °C. Protein was precipitated by the addition of 1,800 μl methanol and pelleted by centrifugation at 12,000g for 5 min. Pelleted protein was resuspended in lysis buffer (8M urea, 40 mM 2-chloroacetamide, 10 mM Tris(2-carboxyethyl)phosphine, 100 mM Tris pH 8) and incubated for 10 min at RT before diluting to [urea] < 2M with 50 mM Tris. Trypsin was added at a protein:enzyme ratio of 100:1 and incubated overnight at RT with gentle rocking. After digesting overnight, the solution was adjusted to pH < 2 and desalted with StrataX reverse phase SPE cartridge (Phenomenex). Eluted peptides were dried under reduced pressure and quantified by bicinchoninic acid assay (Pierce Quantitative Colorimetric Peptide Assay, Thermo Fisher Scientific). Peptides were reconstituted in 0.2% formic acid to a concentration of 1 μg/μl, and a 2 μl injection was separated over a 90 min nanoliquid chromatography method using a nanoAcquity UPLC (Waters). Eluting peptides were analyzed a Q-LTQ-OT Tribrid mass spectrometer (Orbitrap Fusion Lumos, Thermo Scientific) following positive mode electrospray ionization. MS1 survey scans were performed in the orbitrap (240K resolution, AGC target – 1e6, 100 ms max injection time). Tandem mass spectra of HCD-generated (25% NCE) product ions were performed in the ion trap (rapid resolution, AGC target – 4e4, 18 ms maximum injection time). Monoisotopic precursor selection and dynamic exclusion (15 s) were enabled. Thermo RAW files were searched against the Uniprot Human Reference Proteome spectral library (downloaded February, 2018; 93,798 forward sequences searched in database) with the MaxQuant (Version 1.6.0.13) quantitative software suite (19). Peptides were searched using full-tryptic cleavage constraints with maximum two missed or nonspecific cleavages. Searches were performed with precursor mass tolerance of 50 ppm and product ion mass tolerance of 0.2 Da. Carbamidomethylation of cysteines was imposed as a fixed modification and oxidation of methionines as a variable modification. ‘Match between runs’ and ‘label-free quantification’ were enabled with a match time window of 0.7 min and minimum ratio count of 1. Search results were filtered to a false discovery of 1% at both the peptide and proteins levels. The mass spectrometry proteomics data have been deposited to the ProteomeXchange Consortium *via* the PRIDE partner repository with the dataset identifier PXD033373 (20).

#### Experimental Design and Statistical Rationale

Discovery proteomics for each oncogene model were analyzed in biological triplicates. MaxQuant parameters and false discovery rate filters for peptide searches are detailed in proteomics methods above.

MaxQuant LFQ intensities were imported into Perseus v1.6.7.0 for processing and statistical analysis using standard procedures (21, 22, 23). First, technical replicates were grouped into biological replicates, and the oncogene *versus* nononcogene conditions were annotated. Data were filtered for contaminants, processed for razor+unique peptides (x > 1), and filtered for valid values in 60 to 70% of technical replicates of least one experimental condition. Missing data were imputed using a normal distribution. Technical replicates were collapsed into biological replicates by computing the mean LFQ value. We proceeded with statistical analysis of the three biological replicates using permutation-based false discovery rate t-tests (250 repetitions) to account for multiple-hypothesis testing (P493–6, LHS, PDAC, OS models) or using *t* tests with Bonferroni adjustments to correct for multiple hypothesis testing (MCF10A models). Thresholds for upregulated and downregulated proteins were *p* ≤ 0.05 and log_2_FC≥ |1|. Gene-set enrichment was performed using REACTOME bioinformatics tools (24).

### Cellular Treatments: Nucleic Acid, cGAMP, anti-hIFNβ, hIFNβ, GSK8612, MEKi

Twenty-four hours before treatments, cells were counted and plated at equal densities.

For nucleic acid and cGAMP stimulation experiments, cells were plated, and transfections were carried out in reduced-serum Opti-MEM media (UCSF Cell Culture Facility). PolyI:C (InvivoGen, Cat# tlrl-picw), dsDNA harvested from salmon (Thermo Fisher Scientific, Cat# 15632011), or cGAMP (ApexBio, Cat# B8362) were transfected using PEI (Polyplus-transfection, Cat# 115–010) at a 4:1 PEI:nucleic acid ratio. For phospho-TBK1 immunoblotting, cells were transfected with 0.5 μg/ml polyI:C, 0.5 μg/ml dsDNA, 1 μM cGAMP, or PEI transfection agent alone for 4 h. For T1IFN transcriptional activation experiments, cells were transfected with polyI:C (0.1 μg/ml nucleic acid for P493–6 and LHS or 0.5 μg/ml nucleic acid for MCF10A) or PEI transfection reagent alone for 4 h. For RNASEL rRNA fragmentation analyses, cells were treated with 0.5 μg/ml polyI:C or PEI transfection reagent alone for 4 h.

To establish an IFNβ antibody blockade, LHS EV and MCF10 EV cells were treated with 4 μg/ml anti-hIFNβ (Invivogen, Cat# mabg2-hifnb-3) or PBS for 16 h at 37 °C. For the IFN-response assay and SARS-CoV-2 pretreatment rescue experiment, cells were treated with 500U/ml hIFNβ (STEMCELL Technologies, Cat# 78113.1) or PBS for 16 h at 37 °C.

To inhibit TBK1, LHS EV cells were dosed with 50 μM GSK8612 (MedKoo, Cat# 555464) or DMSO, and proteomic perturbations were determined after 48 h at 37 °C.

For MEK inhibitor studies, cells were treated with 2 μM PD0325901 (Selleck Chemicals, Cat# S1036) or DMSO vehicle and harvested for RNA extraction or immunoblot after 18 h.

### Cloning and Engineering OAS2 Overexpression Cell Lines

OAS2 protein sequence was codon optimized for homo sapiens and purchased as two overlapping gene blocks from Twist Biosciences. EF-1a-driven overexpression plasmid pCDH-EF1-FHC was a gift from Richard Wood (Addgene, Cat# 64874) and was used as the lentiviral backbone for transgene delivery (25). pCDH was opened by digestion with NotI (New England Biosciences, Cat# R3189S), and Gibson assembly was used to insert the overlapping gene fragments into the open backbone. Two constructs were created containing either puromycin or hygromycin resistance cassettes because LHS MYC cells were previously engineered using hygromycin resistance, and MCF10A AKT was engineered using puromycin resistance.

Lentiviral vectors were transfected using Fugene (Promega, Cat# E2311) into HEK293T cells. Cells were maintained at 37 °C for 72 h to permit viral production. Viral supernatants were filtered and added to plated LHS MYC and MCF10A AKT cells. To increase transduction efficiency, cells treated with lentivirus were centrifuged at 1000*g* for 3 h. Cells were subsequently incubated at 37 °C for 24 h. Then viral transduction solution was washed out with PBS and replaced with fresh media. After an additional 24 h at 37 °C, cells were treated with 5 μg/ml puromycin (Sigma Aldrich, Cat# P9620) or 200 μg/ml hygromycin (Thermo Scientific, Cat# 10687010) to select for transgene expression. Media changes continued to be dosed with antibiotics for 2 weeks to select for cells with stable transgene incorporation, at which point knock-ins were validated by OAS2 Western blot and qPCR amplification of the transgene transcript.

### RNA Extraction, cDNA Preparation, and qPCR Reactions

RNA was extracted and purified using either Qiagen RNeasy (Cat# 74104) or IBI Scientific Tri-isolate RNA Pure kits (Cat# IB47632) according to respective manufacturer guidelines. For qPCR assays, RNA was DNAse-treated and converted to cDNA using Quantitect Reverse-Transcription kit (Qiagen, Cat# 205311) according to manufacturer protocols. qPCR reactions were performed using SYBR Select Master Mix (Thermo Scientific, Cat# 4472908). For most transcripts of interest, primer conditions were 250 nM and Tm was 60 °C, however SARS-CoV-2 viral N and E gene transcripts were assayed using 400 nM primer and Tm of 58 °C. Primer sequences are reported in supplemental Table S4 (Integrated DNA Technologies). Fluorescent emissions were detected using Bio-Rad CFX Connect qPCR instrument. Data were analyzed using ΔΔCT method (26).

### OAS-RNASEL RNA Fragmentation Analysis

Total cellular RNA, consisting mostly of rRNA, was prepared with the RNA ScreenTape reagents (Agilent, Cat# 5067-5576, 5067-5577, 5067-5578,) according to manufacturer protocols. Capillary electrophoresis assays were performed and analyzed using the Agilent 4200 TapeStation System and software. The change in RIN^e^ value between unstimulated and polyI:C transfected conditions was calculated for each cell type. To compare oncogene and nononcogene cells, this change in RIN^e^ value was subtracted [Oncogene – EV] and is reported as ΔRIN^e^.

### Authentic SARS-CoV-2 Infection in BSL-3

SARS-CoV-2 from a clinical specimen at UCSF was isolated, propagated, and plaqued on Huh7.5.1 cells overexpressing angiotensin-converting enzyme 2 (ACE2) and transmembrane serine protease 2 (TMPRSS2) (27). Viral titers were determined using standard plaque assays (28). All work involving live SARS-CoV-2 was performed in the CDC/USDA-approved Biosafety Level 3 (BSL-3) facility at the University of California, San Francisco, in accordance with institutional biosafety requirements.

Twenty-four hours prior to SARS-CoV-2 infection, LHS and MCF10A cells were counted and plated in 24-well cell culture plates at equal densities avoiding overseeding that could disrupt a uniform monolayer. Infection was carried out as previously described (29). Immediately before infection, one well for each cell line was trypsinized to count the number of cells per well. Cells were washed in PBS and infected with SARS-CoV-2 at multiplicity of infection (MOI) of 0.1 (LHS cells) or MOI 1.0 (MCF10A cells). After 1 h, the viral inoculum was removed, cells washed in PBS, and 1 ml of complete culture media added to each well. Plates were then incubated at 37 °C/5% CO2 for 24 h. After infection, supernatants were removed, and the cells washed twice with PBS before being lysed in TRIzol for total RNA extraction.

### Immunoblotting

Cells were lysed with RIPA (Millipore Sigma, Cat# 20-188) containing protease inhibitor (Merck, Cat#11836170001) and phosphatase inhibitor (Sigma Aldrich, Cat# 04906845001). Protein gels were transferred to polyvinylidene difluoride membrane using iBlot two instrument and consumables (Thermo Fisher Scientific, Cat# IBI21001, IB24001). Membranes were blocked with 5% BSA for 1 h, primary antibodies were incubated overnight at 4 °C, and appropriate secondary antibodies (LI-COR Biosciences, Cat# 926–32211, 926–68070) were stained for 1 h at room temperature. Blots were imaged using LI-COR Odyssey CLx scanner and processed using Image Studio Lite. Signal for OAS2 knock-in MCF10A AKT cells was too low to quantify by fluorescence and was instead imaged using HRP chemiluminescence (Cell Signaling Technology, Cat# 7076S and 7074S).

The following antibodies were used at recommended manufacturer dilutions: phospho(Ser172)-TBK1 (Cell Signaling Technology, Cat# 5483T), TBK1(Cell Signaling Technology, Cat# 3504T), ACE2 (Cell Signaling Technology, Cat# 4355T), TMPRSS2 (Invitrogen, Cat# MA5-35756), OAS2 (Thermo Fisher Scientific, Cat# TA802770), MYC (Cell Signaling Technology, Cat# 9402S), and ACTINβ (Cell Signaling Technology, Cat# 3700S, 4970S).

### siRNA Transfections

For siRNA experiments, negative control siRNA (Thermo Scientific, Cat# AM4611/Thermo Scientific, Cat# 4390843) or target siRNA (MYC/GAPDH) (Cell Signaling Technology, Cat# 6341S/Thermo Scientific, Cat# 4390849) were transfected with lipofectamine (Thermo Scientific, L3000–008) per standard protocols (no P3000 reagent was used for RNA transfection). The final concentration was 20 nM siRNA, except for OS152 and OS186 cell lines, which were respectively treated with 40 nM and 80 nM negative control/MYC/GAPDH siRNA. Downstream qPCR analyses were performed 48 h posttransfection.

## Results

### Integrative Proteomic Analysis of Cells Expressing Driver Oncogenes Identified Massive Suppression of T1IFN and Antiviral Response Pathways

We used label-free whole cell proteomics to characterize the effect of MYC overexpression for two isogenic MYC models. P493-6 cells are an isogenic model of Burkitt’s lymphoma and overexpress MYC on a tetracycline-repressible promotor (30). P493-6 cells were treated in the presence or absence of tetracycline to generate low or high MYC expression cell lines, respectively. As a second isogenic model, LHS-PrEC (LHS) prostate epithelial cells were engineered with a MYC overexpression plasmid or an empty vector (EV) control (18). We additionally tested two PDX cell lines of metastatic OS carrying high MYC copy amplification (OS152 and OS186) and compared these to normal human fetal osteoblasts (hFOB 1.19) (10). In total, the cell lines span lymphocyte, epithelial, and mesenchymal cancer subtypes providing a broad cellular view of MYC overexpression.

**Fig. 1:**
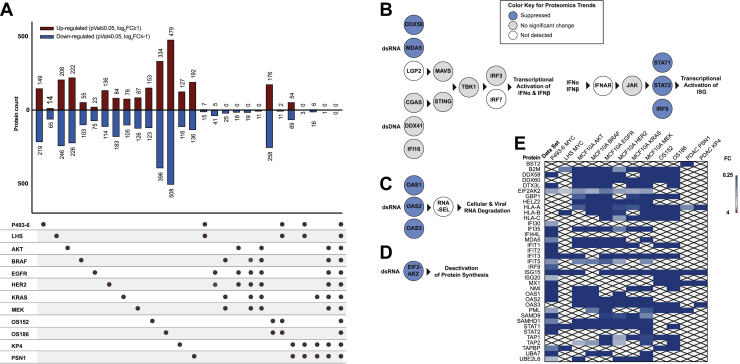
**Expression of oncogenes causes bidirectional remodeling of cellular proteome and reveals strong downregulation of IFN-inducible antiviral pathways.** All mass spectrometry data represent three biological replicates for each cell line. *t* test significance was corrected for multiple hypothesis testing as described in Experimental Procedures. *A*, upset plot summarizing intersections of proteomics results. Thresholds for upregulation and downregulation were *p* ≤ 0.05, log_2_FC≥ |1|. *B–D*, schema of antiviral pathways are colored to represent general trends in proteomics. Proteins that were suppressed in MYC or signal transduction oncogene models are *dark blue*. Proteins that were not dramatically changed in MYC or signal transduction oncogene models are *gray*. Proteins that were not detected in any dataset are *white*. *B*, pathway schema for T1IFN response. The dsRNA and dsDNA sensors drive cascades controlling transcription of IFNα and IFNβ. Secreted IFNα and IFNβ stimulate autocrine and paracrine signaling by binding to the interferon receptor. This activates JAK/STAT signaling leading to formation of the ISGF3 complex that regulates transcription of hundreds of ISGs. *C*, pathway schema for OAS-RNASEL system. dsRNA sensors OAS1-3 catalyze the synthesis of 2′-5′ oligoadenylate chains, ligands to latent RNASEL. Activated RNASEL indiscriminately cleaves cellular and viral RNA. *D*, pathway schema for EIF2AK2 (PKR) activation. EIF2AK2 is activated by dsRNA and phosphorylates EIF2A to inhibit protein synthesis. *E*, heatmap demonstrating decreased protein expression of over 35 ISGs (with p ≤ 0.05). dsRNA, double-stranded RNA; dsDNA, double-stranded DNA; IFN, interferon; ISG, interferon-stimulated gene; T1IFN, type 1 interferon.

Mass spectrometry detected 3579, 3449, and 4235 proteins for the P493-6, LHS, and OS MYC models, respectively, and showed that overexpression of the MYC oncogene causes bidirectional changes to hundreds of proteins (Fig. 1*A* and supplemental Table S1, ProteomeXchange identifier PXD033373). While expression levels of individual proteins differed among the cell lines, gene set enrichment for each dataset harmonized at the pathway level as previously noted for MYC overexpression cell surface proteomes(18). For example, metabolism and ribosome biogenesis pathways classically connected to MYC tumorigenesis were upregulated at the systems level (supplemental Table S2) (6, 31). As the LHS, P493-6, and OS have distinct cellular backgrounds, the overlap of individual protein targets was more moderate. The two isogenic MYC models, P493-6 and LHS, shared a set of seven upregulated proteins (*p* ≤ 0.05, log_2_FC≥1), and two of these proteins were also significantly upregulated in the OS PDX cell lines. Both isogenic MYC cell lines also commonly downregulated 15 proteins (*p* ≤ 0.05, log_2_FC≤-1), and 11 of these proteins were significantly decreased in the OS PDX cell lines (Fig. 1*A*). Strikingly, the proteins and pathways ubiquitously suppressed by MYC in all four isogenic and PDX models converged on T1IFN and antiviral pathways (supplemental Tables S2 and S3). For individual isogenic and PDX systems, MYC expression significantly downregulated respective combinations of up to 28 interferon and antiviral effectors, and these represented four out of the 11 proteins commonly suppressed across all four MYC models. Moreover, the changes for these proteins were some of the most dramatic in the dataset, most ranging from 4-fold to over 200-fold reduced.

To assess changes induced by proliferative signal transduction oncogenes—the tyrosine kinases EGFR and HER2 or the down-stream effectors AKT, KRAS, BRAF, and MEK—MCF10A cells were engineered to overexpress HER2 or to express the common, constitutively active oncogenic forms of KRAS^G12V^, EGFR^L858R^, BRAF^V600E^, MEK^S218D/S222D^, or myristoylated AKT (17). The oncogenic cells and comparator control cells expressing the EV were characterized by label-free whole cell proteomics that detected 5292 proteins for each isogenic model. The data are reported in ProteomeXchange with identifier PXD033373 (supplemental Table S1). Each oncogene caused large upregulation and downregulation for hundreds of proteins (Fig. 1*A*). Although these oncogenes neighbor one another in signal transduction pathways, there was no overlap at the individual protein level, suggesting differences in the specific perturbations that each drives (supplemental Table S2) (17, 32, 33). As with MYC, these differences aligned when viewed at the gene-set level underscoring effective functional redundancy. Commonly upregulated pathways centered on signaling cascades (such as EGFR, PI3K, and Rho GTPase) as well as cell cycle and mitotic processes (supplemental Table S2).

Most strikingly, expression of each signal transduction oncogene caused dramatic downregulation of scores of T1IFN response and antiviral pathway proteins (between 21 and 28 proteins in each dataset); these pathways were also significantly enriched by gene set analysis for each proteomics dataset (supplemental Table S3). In total, only 11 proteins were commonly suppressed (*p* ≤ 0.05, log_2_FC≤-1) for all six proliferative oncogenes, and the majority of these proteins (6/11) were effectors of T1IFN and antiviral response pathways (Fig. 1*A*).

Finally, protein expression profiles for two tissue-derived PDAC cancers, KP4 and PSN1, compared to normal human pancreatic ductal epithelial cells were characterized by label-free whole cell proteomics. Raw data are presented in ProteomeXchange with identifier PXD033373 (supplemental Table S1). KP4 and PSN1 belong to the most aggressive basal (quasi-mesenchymal) subtype of PDAC tumors (34, 35). KP4 and PSN1 are driven by KRAS^G12D^ and KRAS^G12R^ mutations, respectively, as well as amplification of MYC (supplemental Fig. S1*A*) (13, 34, 36, 37, 38). Proteomics demonstrated decreased T1IFN and antiviral response machinery in the basal PDAC models, and gene set analysis identified significant interferon pathway suppression (supplemental Tables S2 and S3). Of over 3300 targets detected by proteomics, 54 and 69 proteins were commonly upregulated or downregulated, respectively, in both PDAC cell lines. Four of these were downregulated antiviral proteins (*p* ≤ 0.05, log_2_FC≤-1), three of which were also downregulated in the isogenic KRAS^G12V^ model. This included HLA-A, which was the only protein found commonly dysregulated across the six isogenic models for proliferative oncogenes and both PDAC cell lines.

The proteomics results are depicted by a color-coded schema to contextualize the dysregulation of three different antiviral pathways (Fig. 1, *B*–*D*). These antiviral systems mitigate invading viral pathogens through designated sensor proteins that detect either dsRNA or dsDNA viral genomes or replication intermediates in the cytosol (39). One particular function of nucleic acid sensors is to initiate signaling cascades that activate transcription of T1IFN: IFNα and IFNβ (Fig. 1*B*). In this T1IFN-inducing pathway, the major cytoplasmic dsRNA sensors are DDX58 (RIG-I), MDA5 (IFIH1), and LGP2 (DHX58) and the main cytoplasmic dsDNA sensors are CGAS, DDX41, and IFI16. Ligand-activated dsRNA and dsDNA sensors signal through the adaptor proteins MAVS and STING (TMEM173), respectively. Downstream these separate pathways converge on phosphorylation of TBK1, inducing phosphorylation and nuclear translocation of IRF3 and IRF7, that control the transcription of IFNα and IFNβ. IFNα and IFNβ proteins are secreted from cells and in autocrine and paracrine fashions bind the interferon receptor (IFNAR, with IFNAR1 and IFNAR2 subunits) causing signal transduction that drives formation of the ISGF3 complex (IRF9, STAT1, and STAT2). ISGF3 activates transcription of hundreds of interferon-stimulated genes (ISGs) that coordinate the cellular antiviral defense *via* mitigating viral entry, replication, transcription, and translation processes (39, 40). In a second major antiviral system, OAS proteins (OAS1, OAS2, and OAS3) are sensors activated by cytosolic dsRNA to catalyze the production of 2′-5′ linked oligoadenylates that activate latent RNASEL (Fig. 1*C*). Activated RNASEL indiscriminately cleaves cellular RNA to obstruct the viral replication cycle (40, 41). Finally, one additional dsRNA sensor, EIF2AK2 (PKR), negatively regulates translational machinery to prevent viral protein synthesis (40) (Fig. 1*D*).

Remarkably, all seven oncogenes significantly downregulated proteins acting in these three major antiviral response pathways (Fig. 1, *B*–*D*). These repressed effectors are also well-annotated ISGs, controlled by interferon-stimulated response elements (ISRE) in their gene regulatory regions. Moreover, there were large magnitudes of suppression (most between 4-fold and over 200-fold) of more than 35 other ISGs including HLA, B2M, and TAP1/2 proteins that are involved in antigen presentation, STAT proteins, ISG15, MX1, and IFIT3 (Fig. 1*E*) (40, 42). The pronounced and global depletion of ISGs implicated that T1IFN could be central in disseminating an ISG-suppressed phenotype in cells expressing oncogenes.

### Depletion of MYC and Inhibition of MAPK Signaling Validates Their Regulation Over Interferon and Antiviral Pathways in PDAC and OS Tumor-Derived Models

Systematic suppression of ISGs was identified for tumor-derived OS and PDAC cell lines when compared to normal cell lines. In addition to showing the phenotype in cancer-derived cells, we used siRNA knockdown of MYC and MAPK inhibitor treatments as alternative approaches to validate that depletion of oncogenes and inhibition of their signaling reverses these effects on interferon and ISG expression.

First, knockdown of endogenous MYC in the LHS parental cells (from which EV and MYC are derived) produced 1 to 2 orders of magnitude increases in transcript levels of IFNβ and a panel ISG (IRF7, OAS2, OAS3, DDX58, STAT1), measured by qPCR (supplemental Fig. S1, *B* and *C*). Thus, depletion of MYC produced the opposite effect of MYC overexpression. These effects also suggest that even normal cellular concentrations of MYC regulate baseline IFNβ and ISG expression. Next, MCF10A cells expressing KRAS oncogene were treated with MEK inhibitor PD0325901 (MEKi). Inhibition of MAPK signaling increased IFNβ transcript levels nearly 10-fold and caused corresponding upregulation of ISGs, validating the effects observed using MAPK oncogene overexpression models (supplemental Fig. S1*H*).

Finally, we directly confirmed that MYC and KRAS oncogenes regulate the interferon and ISG suppression phenotypes in tumor-derived PDAC and OS models using siRNA knockdown of MYC and MEKi treatment. MYC knockdown in KP4 and PSN1 cell lines produced 1 to 2 orders of magnitude increases in IFNβ and ISG transcript levels. Similarly, MYC knockdown in OS152 and OS186 PDX-derived cell lines caused dramatic upregulation of IFNβ and ISG transcript levels, several induced by over three orders of magnitude (supplemental Fig. S1, *B*, *D* and *E*). Additionally, KP4 and PSN1 cell lines expressing mutant KRAS were treated with MEKi. Inhibition of MAPK signaling caused 2-fold to over 20-fold upregulation of IFNβ and ISG transcript levels (supplemental Fig. S1*I*). These isogenic knockdown and pathway inhibition experiments control for genetic complexities between PDAC and OS tumor cells *versus* normal cells, clearly demonstrating that expression of MYC and KRAS oncogenes drives the ISG suppression phenotype that was identified using unbiased proteomics.

### dsRNA Sensing Proteins are Among the Most Dysregulated ISGs Causing Impaired Functional Response to polyI:C Stimulation

**Fig. 2:**
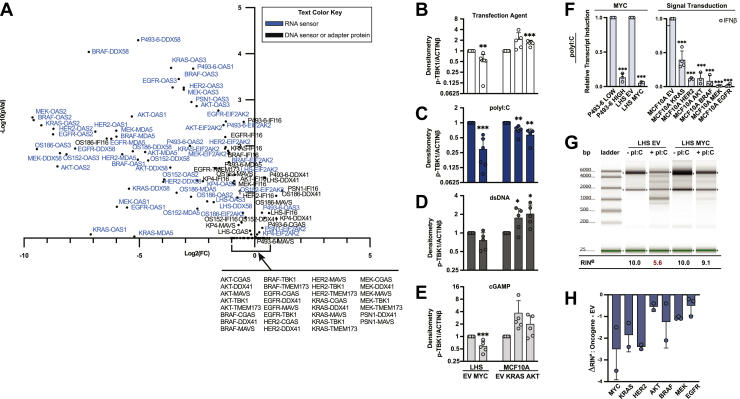
**Oncogenes disproportionately downregulate dsRNA sensing compared to dsDNA sensing pathways at proteomic and functional level.***A*, volcano plot depicts dsRNA sensor, dsDNA sensor, and adaptor protein fold changes and *p* values for oncogene *versus* nononcogene cells. Each data point is labeled by the protein and the associated tumor model. dsRNA sensors are labeled in *blue* text. *B–E*, cells with or without oncogenes were treated with transfection agent alone (*B*) or complexed with polyI:C (*C*), salmon dsDNA (*D*), or cGAMP (*E*). Phosphorylation at Ser172 of TBK1 was immunoblotted. Densities were normalized to the value for respective EV cells for each treatment. Bar graphs represent mean and standard deviation for five biological replicates. Statistics were calculated using Student’s *t* test between EV and oncogene. *F*, cells with or without oncogenes were treated with transfection agent alone or complexed with polyI:C. Transcript level of IFNβ relative to GUSβ reference gene was quantified by qPCR. Extent of IFNβ induction was calculated as the fold change in IFNβ mRNA between polyI:C treatment and transfection agent alone. Data are normalized to induction value of EV. Bar graphs represent mean and standard deviation of at least three biological replicates. Statistics were calculated using Student’s *t* test between EV and oncogene. *G–H*, cells with or without oncogenes were treated with transfection agent alone or complexed with polyI:C and activation of RNASEL was quantified by capillary electrophoresis. *G*, representative capillary electrophoresis experiment for LHS EV and LHS MYC cells. Cleavage of RNA was quantified by RIN^e^ values. *H*, the reported ΔRIN^e^ in bar graphs is the difference between RIN^e^(Oncogene)—RIN^e^(EV). Bar graph reports mean and standard deviation of at least two biological replicates. RIN^e^ values are tabulated in supplemental Fig. S3*G*. ∗*p* ≤ 0.05, ∗∗*p* ≤ 0.005, ∗∗∗*p* ≤ 0.0005. dsRNA, double-stranded RNA; dsDNA, double-stranded DNA; ISG, interferon-stimulated gene; IFN, interferon; T1IFN, type 1 interferon.

Interestingly, the proteomics data showed more dramatic effects on dsRNA than dsDNA sensing pathway proteins. Proteins that sense cytosolic dsRNA were downregulated from 2-fold to over 50-fold. In contrast, the dsDNA sensors or the adaptor proteins MAVS, STING, and TBK1 were either insignificantly or only modestly changed when detected in the proteomics (Fig. 2*A*). The dsRNA sensors DDX58, MDA5, OAS proteins, and EIF2AK2 are well-annotated as ISGs. The dsDNA sensors and adaptor proteins have not been identified as strong ISGs; however, one report found that cGAS was induced by T1IFN in macrophages (43). To test if cGAS is regulated by T1IFN in the cell types used here, the cell lines were treated with 500U/ml IFNβ, and the mRNA levels of a panel of well-annotated ISG—OAS2, OAS3, DDX58, and STAT1—and cGAS were quantified by qPCR. In contrast to the orders of magnitude increases in transcription of strong ISGs, cGAS was not regulated by IFNβ in these cell lines, indicating it may be a weaker or cell-type–specific ISG (supplemental Fig. S2, *A* and *B*) (40, 42, 44, 45). Therefore, consistent with the global suppression of ISGs, we hypothesized that a deactivated T1IFN state in tumor cells exerts greater impact on T1IFN-regulated dsRNA sensors than other pathway elements with weaker or absent ISRE.

Based on the significantly reduced protein levels of dsRNA sensors but similar levels of dsDNA sensors, we predicted that there would be distinct functional consequences to the dsRNA sensing compared to the dsDNA sensing signaling pathways for cells expressing oncogenes. The dsRNA and dsDNA sensing cascades that regulate production of T1IFN converge downstream of MAVS and STING at the phosphorylation at Ser172 of TBK1. Therefore, we measured activation of TBK1 in response to dsRNA or dsDNA ligands to determine the relative nucleic acid sensor function between cells expressing oncogenes and EV. To assess dsRNA sensing, cells were stimulated with the dsRNA mimetic polyinosinic-polycytidylic acid (polyI:C). Low molecular weight polyI:C was chosen as it is the optimal length for DDX58 activation; however, MDA5 requires longer dsRNA ligands (46). To evaluate dsDNA sensing, cells were treated with dsDNA from salmon that was purchased presheared to average 1000 bp, within the length range for optimal cGAS activation (47). STING protein levels were comparable in the MCFF10A signal transduction oncogene models but below mass spectrometry detection limits in LHS and P493-6 cells, so in addition cells were treated with 2′3′-cyclic GMP-AMP (cGAMP), the cyclic dinucleotide activator of STING synthesized by cGAS, to directly examine STING function (48).

**Fig. 3:**
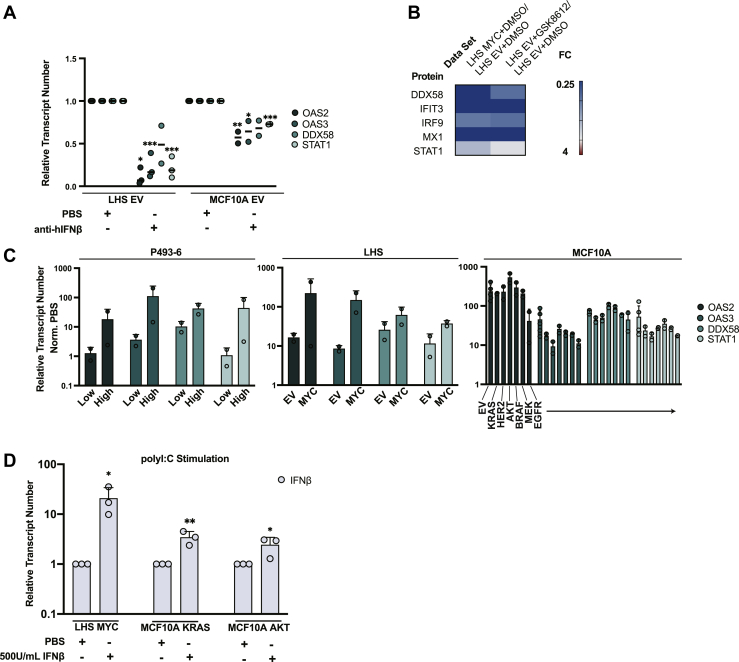
**Reduced autocrine activity of IFN**β **produces a state of low ISG expression**. *A*, LHS EV and MCF10A EV cells were treated with anti-hIFNβ or PBS. Transcript levels of ISGs relative to GUSβ reference gene were quantified by qPCR. Values were normalized to PBS treatment. Data represent two or three biological replicates. Statistics were calculated using Student’s *t* test between PBS and anti-hIFNβ treatment. *B*, LHS EV cells were treated with 50 μM GSK8612 or vehicle (DMSO) and characterized by label-free proteomics. Heatmap compares fold change values for a set of ISGs (with *p* ≤ 0.05) for (*left column*) LHS MYC *versus* LHS EV and (*right column*) LHS EV + GSK8612 *versus* LHS EV + DMSO. Heatmap represents two biological replicates. *C*, isogenic oncogene models were stimulated with 500U/ml hIFNβ or PBS. mRNA levels of ISG relative to GUSβ reference gene were quantified by qPCR. Bar graphs summarize fold change between PBS and IFNβ treatment for each cell line, and report mean and standard deviation of biological duplicates. *D*, cells expressing MYC, KRAS, and AKT oncogenes were pretreated with 500U/ml hIFNβ or PBS and subsequently stimulated with transfection agent alone or complexed with polyI:C. Transcript level of IFNβ relative to GUSβ reference gene was quantified by qPCR. Extent of IFNβ induction was calculated as the fold change in IFNβ mRNA between polyI:C treatment and transfection agent alone, and values were normalized to the PBS treatment. Bar graphs represent mean and standard deviation for three biological replicates. Statistics were calculated using Student’s *t* test between PBS and hIFNβ treatments. ∗*p* ≤ 0.05, ∗∗*p* ≤ 0.005, ∗∗∗*p* ≤ 0.0005. dsRNA, double-stranded RNA; dsDNA, double-stranded DNA; EV, empty vector; ISG, interferon-stimulated gene.

Cells expressing MYC, KRAS, and AKT oncogenes (selected to represent the three major oncogenic axes) and EV were stimulated with transfection agent alone or transfection agent complexed with polyI:C, dsDNA, or cGAMP, and the phosphorylation of TBK1 was quantified by immunoblot. Mass spectrometry data and immunoblots showed equivalent levels of total TBK1 indicating no change in protein expression (supplemental Fig. S3*A*). For the transfection agent control, there were similar levels of baseline phosphorylation of TBK1 for the MCF10A cells expressing EV, KRAS, and AKT; however, substantial hypophosphorylation of TBK1 for LHS cells overexpressing MYC compared to EV (53% of EV levels) (Figs. 2*B* and S3*B*). When stimulated with polyI:C, cells expressing oncogenes had significantly reduced TBK1 activation when normalized to the level for EV (approximately 29%, 70%, and 57% of EV level for LHS MYC, MCF10A KRAS, and MCF10A AKT respectively) (Figs. 2*C* and S3*C*). In contrast, the cells expressing KRAS and AKT produced similar or increased levels of phospho-TBK1 compared to MCF10A EV when treated with dsDNA and cGAMP (Figs. 2, *D* and *E* and S3, *D* and *E*). The LHS MYC cells had reduced levels of phospho-TBK1 compared to LHS EV when stimulated with dsDNA and cGAMP (76% and 58% of EV levels respectively); however, these differences were not as dramatic as the larger effect produced by polyI:C treatment and likely residual of the hypophosphorylation observed at baseline (Figs. 3, *D*, *E* and S2, *D* and *E*). The desensitization to polyI:C stimulation demonstrated for MYC, KRAS, and AKT oncogenes implicated dysfunction in dsRNA sensing upstream of TBK1 that was consistent with the proteomics results.

Based on the impaired activation of TBK1 with dsRNA stimulation, we expected that downstream activation of T1IFN transcription would be correspondingly diminished in cells expressing driver oncogenes compared to EV. To evaluate this functional effect, all eight isogenic oncogene models were stimulated with polyI:C, and the transcriptional activation of T1IFN was quantified by qPCR using pan-IFNα and IFNβ primers. T1IFN induction was calculated as the fold change in transcript levels for transfection agent alone treatment *versus* transfection agent complexed with polyI:C treatment. To evaluate the relative dsRNA sensing responses for cells expressing oncogenes *versus* EV, we normalized the fold change value for cells expressing oncogenes to the fold change value for the corresponding EV control (Figs. 2*F* and S3*F*). When compared to nononcogene controls, P493-6 and LHS cells overexpressing MYC had 4- to 15-fold decreased induction of T1IFN when treated with polyI:C (Figs. 2*F* and S3*F*). MCF10A cells expressing signal transduction oncogenes were similarly desensitized to polyI:C when compared to MCF10A EV, exhibiting 10-fold to over 100-fold reduced transcriptional activation (Figs. 2*F* and S3*F*). Compared to MCF10A EV, cells expressing AKT oncogene showed dramatically reduced IFNβ induction; however, the induction of IFNα was not statistically different (Figs. 2*F* and S3*F*). This result might reflect compounded noise from measuring 13 IFNαs collectively. The 10-fold decrease in IFNβ induction was the dominating effect and supported the observation that cells expressing signal transduction oncogenes had dysfunctional responses to polyI:C. Overall, cells expressing oncogenes had reduced phosphorylation of TBK1 and attenuated induction of T1IFNs when stimulated with polyI:C. Furthermore, this significant result was demonstrated using a ligand that predominantly activates DDX58, and we predict even greater desensitization to dsRNA when the full effect of other suppressed dsRNA sensors like MDA5 is measured.

Next, we evaluated a second dsRNA sensing pathway, the OAS-RNASEL system, that is regulated by the dsRNA sensors OAS1, OAS2, and OAS3. As described, OAS proteins are strongly regulated by T1IFN and are up to two orders of magnitude suppressed in the proteomics data. We hypothesized that polyI:C stimulation would result in low activation of the OAS-RNASEL system in cells expressing oncogenes due to reduced baseline and interferon-induced OAS protein expression. To quantify RNASEL activation, cells expressing oncogenes and nononcogene controls were treated with polyI:C, cellular RNA was extracted, and the extent of RNASEL-driven RNA cleavage was analyzed by capillary electrophoresis. A representative RNA fragmentation trace for the LHS model is shown in Figure 2*G*. Both unstimulated LHS EV and LHS MYC cells had intact RNA as seen by the two dominant rRNA bands matching 18S and 28S subunits, and the corresponding RNA-integrity values (RIN^e^) were the maximum, 10. When stimulated with polyI:C, the RNA banding pattern for LHS EV visually became more fragmented than that of the LHS MYC cells. This was quantified by the lower RIN^e^ number of 5.6 for LHS EV cells compared to 9.1 for LHS MYC cells. The increased RNA degradation in nononcogene cells suggested efficient activation of the OAS-RNASEL pathway, whereas cells overexpressing MYC failed to elicit the equivalent response. Similarly, ΔRIN^e^ [Oncogene-EV] calculations for each oncogene *versus* EV are summarized in Figure 2*H* (RIN^e^ values reported in supplemental Fig. S3*G*) and indicated reduced activation of RNASEL in cells expressing oncogenes compared to respective EV cells.

### Downregulated ISG Expression is Due to Diminished Production of T1IFN

T1IFN produced by cells is important for autocrine regulation of ISGs (39, 40, 49). The global basal suppression of ISGs indicated possible dysregulation of T1IFN expression and autocrine signaling. To test if secreted IFNβ could have this effect, LHS and MCF10A cells expressing EV were treated with an antibody to neutralize IFNβ activity (anti-hIFNβ) or vehicle (PBS), and transcription of a representative set of ISG was profiled by qPCR. Repression of OAS2, OAS3, DDX58, and STAT1 was recapitulated by IFNβ antibody blockade (Fig. 3*A*). In a second approach, LHS EV cells were treated with the specific TBK1 inhibitor GSK8612 or vehicle (DMSO) to block baseline cellular production of IFNβ. Whole cell proteomics of vehicle compared to GSK8612 treatment determined that inhibition of endogenous TBK1 phenocopied the ISG perturbations of oncogenic cells (Fig. 3*B* and supplemental Table S1, ProteomeXchange with identifier PXD033373). In particular, dsRNA sensor DDX58 as well as other strong ISGs such as MX1 and IFIT3 were downregulated 3.1-, 74.6-, and 8.2-fold, respectively, at the TBK1 inhibitor concentration tested (Fig. 3*B*).

We next profiled endogenous levels of T1IFN to further examine the dysregulation of T1IFN expression and autocrine signaling. However, baseline cellular and secreted T1IFN levels for oncogene and nononcogene LHS and MCF10A cell lines were too low to be quantified in cell lysates or conditioned media by commercial ELISA kits. Though baseline T1IFN concentrations could not be determined, treatment with exogenous hIFNβ rescued ISG expression in oncogenic cells. Oncogene and nononcogene cells were treated with 500U/ml hIFNβ or vehicle (PBS), and transcriptional activation of OAS2, OAS3, DDX58, and STAT1 was quantified by qPCR. ISG induction was determined by calculating the transcript fold change between hIFNβ and PBS treatments (Fig. 3*C*). ISG fold changes between hIFNβ and PBS treatment were approximately the same for MCF10A cells expressing signal transduction oncogenes or EV (Fig. 3*C*). LHS and P493-6 cells overexpressing MYC seemingly produced even higher ISG transcriptional responses than nononcogene controls (Fig. 3*C*). However, in a second analysis of the same data, the transcript levels for LHS and P493-6 models were normalized to the value of PBS-treated nononcogene cells (supplemental Fig. S4, *A* and *B*). This demonstrated that the apparent increase in ISG transcription in oncogene cells was likely the combined effect of two to three orders of magnitude reduced transcript levels at baseline and a maximum threshold of IFN and ISG activation before triggering well-described negative feedback pathways (50, 51).

While whole-cell proteomics did not detect T1IFN receptor subunits, previous extracellular-enriched surface proteomics performed on these cell lines identified that IFNAR1 and IFNAR2 expression were generally unchanged in MCF10A isogenic models (17). Despite suppression of interferon-regulated components of the ISGF3 complex, cells expressing oncogenes were poised to re-activate ISG transcription in response to exogenous T1IFN. These results indicated that impaired T1IFN production perpetuates the suppressed antiviral phenotype, while the autocrine/paracrine response arm remains functional.

Finally, the functional rescue by exogenous interferon was tested. Cells expressing MYC, KRAS, and AKT oncogenes were pretreated with either 500U/ml hIFNβ or PBS and subsequently stimulated with polyI:C. The fold change in IFNβ transcript levels between transfection agent alone and transfection agent complexed with polyI:C treatment was calculated, and value for interferon pretreated cells was normalized to its corresponding PBS control (Fig. 3*D*). Pretreating cells expressing oncogenes with interferon rescued the response to polyI:C 3- to 10-fold. Taken together, the phenotyping, autocrine assays, and functional rescue experiments indicated that decreased interferon production prevents autocrine stimulation of antiviral response pathways causing reduced dsRNA sensing in oncogenic cells.

### Cells Overexpressing Oncogenes are More Susceptible to SARS-CoV-2 Infection

**Fig. 4:**
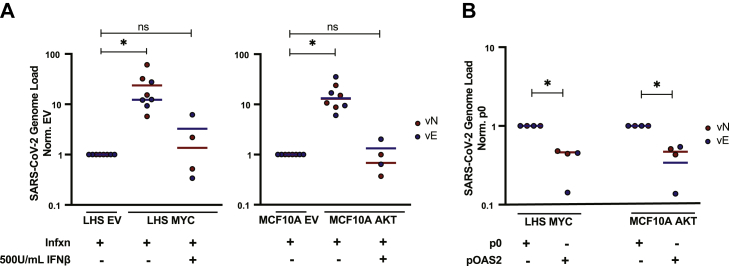
**Oncogene transformed cells are more easily infected with the RNA virus but become more resistant when primed with IFNβ or engineered to re-express OAS2.***A*, LHS EV/LHS MYC cells and MCF10A EV/MCF10A AKT cells with or without 500U/ml IFNβ pretreatment were infected (infxn) with SARS-CoV-2 (MOI 0.1 and 1.0 for LHS and MCF10A respectively—choice in MOI for each cell type was determined in preliminary experiments in supplemental Fig. S5*B*). Cellular RNA was harvested, and viral genome load was quantified by qPCR amplification of viral N (vN) and E (vE) genes relative to cellular GUSβ reference gene. Transcript values were normalized to EV value. Data report two to four biological replicates. Statistics were calculated using Student’s *t* test between EV and oncogene or between EV and oncogene with IFNβ pretreatment. *B*, LHS MYC and MCF10A AKT cells were engineered with stable overexpression of OAS2 (pOAS2) or empty plasmid (p0). pOAS2 and p0 cells were infected with SARS-CoV-2. Cellular RNA was harvested, and viral genome load was quantified. Transcript values were normalized to p0 cell lines. Data represent two biological replicates. Statistics were calculated using Student’s *t* test between cells expressing p0 and pOAS2. ∗*p* ≤ 0.05, ns, not significant. EV, empty vector.

The systematic suppression of T1IFN and antiviral defenses by oncogenes has clinical implications, including potential selective susceptibility to oncolytic and gene therapy viruses. The cumulative impact of reduced T1IFN levels, low ISG expression, and disarmed RNA sensing was interrogated by infecting cells with an RNA virus. We used SARS-CoV-2 because it is a positive-strand RNA virus that generates dsRNA replication intermediates (52). LHS cells overexpressing EV or MYC and MCF10A cells expressing EV and AKT oncogenes were acutely infected with SARS-CoV-2 for 1 h. After infection, virus was washed out, media replaced, and cells were incubated for 24 h to permit viral replication in cells. Cellular RNA was harvested, and viral genome titers were determined by qPCR amplification of viral N (vN) and E (vE) genes (relative to cellular GUSβ). Cells expressing MYC and AKT oncogenes fostered 10- to 20-fold higher viral genome loads compared to respective EV cells treated at equal MOI (Fig. 4*A*). ACE2 and TMPRSS2 are the host receptor and protease that mediate SARS-CoV-2 cellular entry. We immunoblotted these protein levels in LHS EV and MYC cells and MCF10A EV and AKT cells. LHS MYC cells had small increases (less than 2-fold) in ACE2 and TMPRSS2 levels compared to EV cells, and MCF10A AKT cells had similar or decreased expression of ACE2 and TMPRSS2 compared to EV cells, indicating that expression levels of these proteins are not likely causing the dramatic increases in viral titers (supplemental Fig. S5*A*) (53).

To further demonstrate that deactivated antiviral defenses cause increased viral infection (and not ACE2/TMPRSS2 levels or other oncogene effects on biosynthetic or anti-apoptotic pathways), two rescue experiments were performed. In the first experiment, cells were pretreated with 500U/ml hIFNβ for 16 h. IFNβ pretreatment of cells expressing MYC and AKT oncogenes decreased viral titers to nononcogene EV levels, validating that low baseline T1IFN and corresponding low ISG expression specifically caused increased viral infection (Fig. 4*A*). Priming LHS and MCF10A EV with IFNβ did not provide additional defense against viral load (Figs. 4*A* and S5*D*). This is possibly due to already very low levels of infection at baseline because of functional antiviral pathways in nononcogene cells.

In a second rescue experiment, the isolated contribution of OAS-RNASEL system was assessed. Several reports indicate SARS-CoV-2 is sensitive to OAS-RNASEL antiviral defenses, including genome-wide association studies implicating the OAS gene cluster in critically ill patients (52, 54, 55). To determine the consequence of low OAS protein expression, oncogenic cell lines were engineered to stably overexpress OAS2 (pOAS2) or empty plasmid (p0) (supplemental Fig. S5, *E* and *F*). While OAS3 is the primary activator of RNASEL during most viral infections, oncogenic cells failed to express ectopic OAS3 at levels that could be validated by immunoblot (41, 56). Alternatively, we could engineer high levels of OAS2 expression, and when OAS2 is overexpressed there is high activation of RNASEL (41, 57). In cells expressing MYC and AKT oncogenes, OAS2 knock-in partially attenuated (approximately 50%) the viral titers compared to empty plasmid control cell lines (Fig. 4*B*).

Individually, IFNβ pretreatment or OAS2 re-expression could specifically protect cells expressing MYC and AKT oncogenes from viral infection, suggesting oncogene-driven suppression of antiviral defense pathways increases viral susceptibility. Additionally, one consideration is that SARS-CoV-2 encodes multiple viral proteins that block host activation of T1IFNs (58). Based on the impaired activation of T1IFN production in cells expressing oncogenes that we have demonstrated, we hypothesize that there could be even more dramatic tumor cell selectivity for RNA viruses that do not evade the host interferon response.

## Discussion

The isogenic cell line studies here represent a reductionist approach to understanding the impact of well-known driver oncogenes when expressed in immortalized cells. While this oversimplifies oncogenic transformation, these systematic experiments identify molecular and functional changes that are directly regulated by oncogenes and build fundamental understanding that can be applied to diverse and mutationally complex tumors. We confirmed these findings in relevant PDAC- and OS tumor-derived and PDX-derived cell lines.

We utilized unbiased proteomics to evaluate the molecular changes associated with different driver oncogenes and to identify phenotypic convergence that would be relevant to cancer biology or therapeutic strategies. Overall, expression of each oncogene caused large up- and down-perturbations in proteomes, and there was a mixture of uniquely and commonly dysregulated proteins. For the MYC oncogene expressed in B-cell, prostate, and OS models, the distinct regulation highlights dependency on cellular context. The unique set of changes generated by each signal transduction oncogene likely reflects differences in wiring and feedback loops as has previously been observed for the components in the MAPK pathway (32, 33). Despite differences at the individual target level, there was increased overlap when analyzed by gene set analysis that groups proteins by their functional classes. Previous cell surface proteomics studies using these cell lines similarly characterized bi-directional remodeling and a mixture of unique and common proteins that harmonized when viewed by gene set analysis (17, 18).

The most remarkable finding was that oncogenes from distinct signaling axes (MYC, HER2/EGFR, KRAS/BRAF/MEK, and AKT) suppress T1IFN autocrine signaling, which strongly reduces ISG and dsRNA sensor expression. It is interesting that though the endogenous levels of IFNβ are below detection levels by ELISA, it is clearly operating in the normal cells because neutralizing antibodies to it suppress ISG transcription and addition of IFNβ to oncogene cells restored the antiviral expression. This likely reflects the extreme sensitivity of autocrine signaling. The numerous antitumor functions of T1IFN and antiviral effectors are well-known, and immune evasion is a hallmark of cancer (59, 60, 61, 62, 63). Others have found dysregulation of T1IFN and antiviral pathways in a number of advanced and genetically complex cancers, supporting the breadth of the phenotype and its persistence in paracrine tumor microenvironments (59, 60, 64, 65, 66, 67, 68). Here, we expand this understanding by isolating the role of driver oncogenes from other complex mutational lesions and specific cellular contexts and show each oncogene can directly suppress T1IFN and antiviral dsRNA pathways. Further, the interferon suppression signature was the most significant common effect identified using an unbiased and integrative proteomics approach for six signal transduction oncogenes and MYC. These findings emphasize that these pathways may be fundamental in tumor development and immune evasion hallmarks, and support the generality that overactivation of growth and proliferation signaling is immunosuppressive.

The proteomics results indicated that suppression of T1IFN in cells expressing oncogenes has a significant impact on T1IFN-regulated dsRNA sensors but not dsDNA sensors with weaker or absent ISRE. Tumors often carry defects in dsDNA sensing function, for example through genetic and epigenetic repression of cGAS and STING, as well as various mechanisms modulating cGAMP hydrolysis and trans signaling (69, 70, 71, 72, 73). Conversely, STING activation of noncanonical inflammatory pathways has been found to promote epithelial–mesenchymal transition and metastasis in cancers with high chromosomal instability that generate excessive dsDNA in the cytosol (74). It is possible that evasion or activation of dsDNA sensing pathways in tumors is largely shaped by specific tumor contexts, selective pressures, and immune editing that are not captured by reductionist models (48, 69, 70, 75, 76, 77, 78). Our work demonstrates that separate from specific tumor or immune selective factors, oncogenes autonomously downregulate T1IFN expression, causing direct and dramatic consequences to antiviral dsRNA sensors that are strong ISGs.

For each oncogene, we systematically interrogated the functional consequences to dsRNA sensing pathways including T1IFN transcription and OAS activation and the response to interferon. These pathways are critical to several standard cancer therapies. Ionizing radiation, genotoxic drugs, and epigenetic inhibitors require induction of T1IFN and activation of RNASEL to execute cytotoxic and immune activating effects (79, 80, 81, 82, 83, 84). Genotoxic stress and DNA demethylation mount the dsRNA sensor response through upregulated transcription of repetitive, noncoding, and retrotransposon elements that have double-stranded RNA secondary structures (85, 86, 87). In several studies, cells deficient in MAVS, DDX58, OAS proteins, or RNASEL had decreased responses to radiation and epigenetic treatments (80, 81, 82, 83, 87, 88, 89). We demonstrated that prominent oncogenes downregulate DDX58, MDA5, and OAS proteins causing robust deactivation of these dsRNA sensing pathways, which could limit the therapeutic index of radiation, genotoxic, and epigenetic agents. Indeed, the ISG gene signature stratifies radioresistance in breast cancer and is regarded as a radiation-induced biomarker (90). Our discovery that exogenous IFNβ rescued ISG expression in oncogenic models suggests that cotreatment of ionizing radiation, genotoxic drugs, and epigenetic inhibitors with T1IFN could re-sensitize these pathways for broader therapeutic reach. Several studies have reported increased efficacy using combination treatments with interferon (91, 92, 93). However, researchers have found that one mechanism of acquired radioresistance is through selection for insensitivity to interferon. Interestingly, resistant cells fail to transmit interferon signaling but depend on the constitutive expression of unphosphorylated STAT1, which they showed to be a response to chronic interferon stimulation during radiation (94, 95).

Evasion of immune surveillance is a signature of many cancers, and checkpoint inhibitors and adoptive cell therapies are strategies to promote immune cell infiltration (1, 2, 96). T-cell recruitment requires antigen-presentation on major histocompatibility complexes (MHC), which are regulated by interferon. Tumors with low MHC or interferon expression are resistant to these therapies, and a genetic screen specifically identified HLA-A, B2M, TAPBP, TAP1, TAP2, and STAT1 as essential genes for immunotherapy response (97, 98, 99). These are ISGs that were found significantly downregulated by oncogenes in our proteomics. These are important considerations for therapies that rely on native immune cell recruitment, which other investigators have shown become more effective when cotreated with interferon (100, 101). CAR T-cells are engineered to recognize upregulated surface proteins and could be advantageous for targeting tumor cells that might downregulate MHC complexes through repressed interferon.

We found that cells expressing oncogenes have increased viral vulnerability. While our experiments tested SARS-CoV-2 as proof-of-concept, the selective viral susceptibility of tumor cells could be relevant to oncolytic and gene therapy viruses. There has been extensive research and ongoing clinical trials for viral-based cancer therapeutics that exploit tumor-intrinsic pro-proliferation and anti-apoptosis pathways and immune-privileged microenvironments (102, 103, 104). Other researchers have demonstrated the efficacy of virally targeting tumors with specific defects that downregulate innate immune signaling, and studies using the proviral drug sunitinib implicated that inhibition of OAS-RNASEL and EIF2AK2 enhances efficacy of oncolytic virus (66, 105, 106, 107, 108, 109). Our experiments showed that dramatic desensitization of dsRNA sensing pathways and increased susceptibility to RNA viral infection are general effects of oncogenes that could be broadly leveraged using virotherapy. Further, our functional discoveries would suggest that a virus that does not encode proteins to evade host antiviral and interferon response pathways could be highly tumor-selective by exploiting attenuated T1IFN production in tumor cells compared to healthy tissues (110, 111).

It is significant that seven driver oncogenes suppress interferon and harmonize at phenotypic and functional levels. PDAC and OS are two highly lethal diseases, and for many other cancers low interferon and ISG expression are also indicative of aggressive and drug-resistant subtypes (10, 13, 14, 59, 60, 64, 79). We hope these molecular and functional studies help inspire therapeutic development for these currently undruggable and refractory cancers.

## Data Availability

The mass spectrometry proteomics data have been deposited to the ProteomeXchange Consortium *via* the PRIDE partner repository with the dataset identifier PXD033373.

## Supplemental data

This article contains supplemental data (112, 113, 114, 115, 116, 117, 118, 119).

## Conflict of interest

The authors declare no competing interests.
