# Supplementary material for: Discovery Proteomics Analysis Determines That Driver Oncogenes Suppress Antiviral Defense Pathways Through Reduction in Interferon-β Autocrine Stimulation
Source: Mol Cell Proteomics. 2022 May 18;21(7):100247. doi: 10.1016/j.mcpro.2022.100247 (PMC9212846; doi:10.1016/j.mcpro.2022.100247)
Supplement: Supplemental data [file mmc1.pdf]

Supplemental Data

Proteomics Data Set	REACTOME FDR Interferon alpha/beta signaling
P493-6	8.44E-15
LHS	4.22E-15
AKT	2.55E-15
BRAF	3.55E-15
EGFR	3.29E-15
HER2	2.78E-15
KRAS	4.44E-15
MEK	4.55E-15
OS152	9.1E-15
OS186	9.4E-15
KP4	8.22E-15
PSN1	1.19E-14

Table S3. Gene-set enrichment analysis shows type 1 interferon pathways are suppressed. Gene set enrichment was performed using REACTOME bioinformatics tools. FDR for Interferon alpha/beta signaling pathway was tabulated for each oncogenic model.

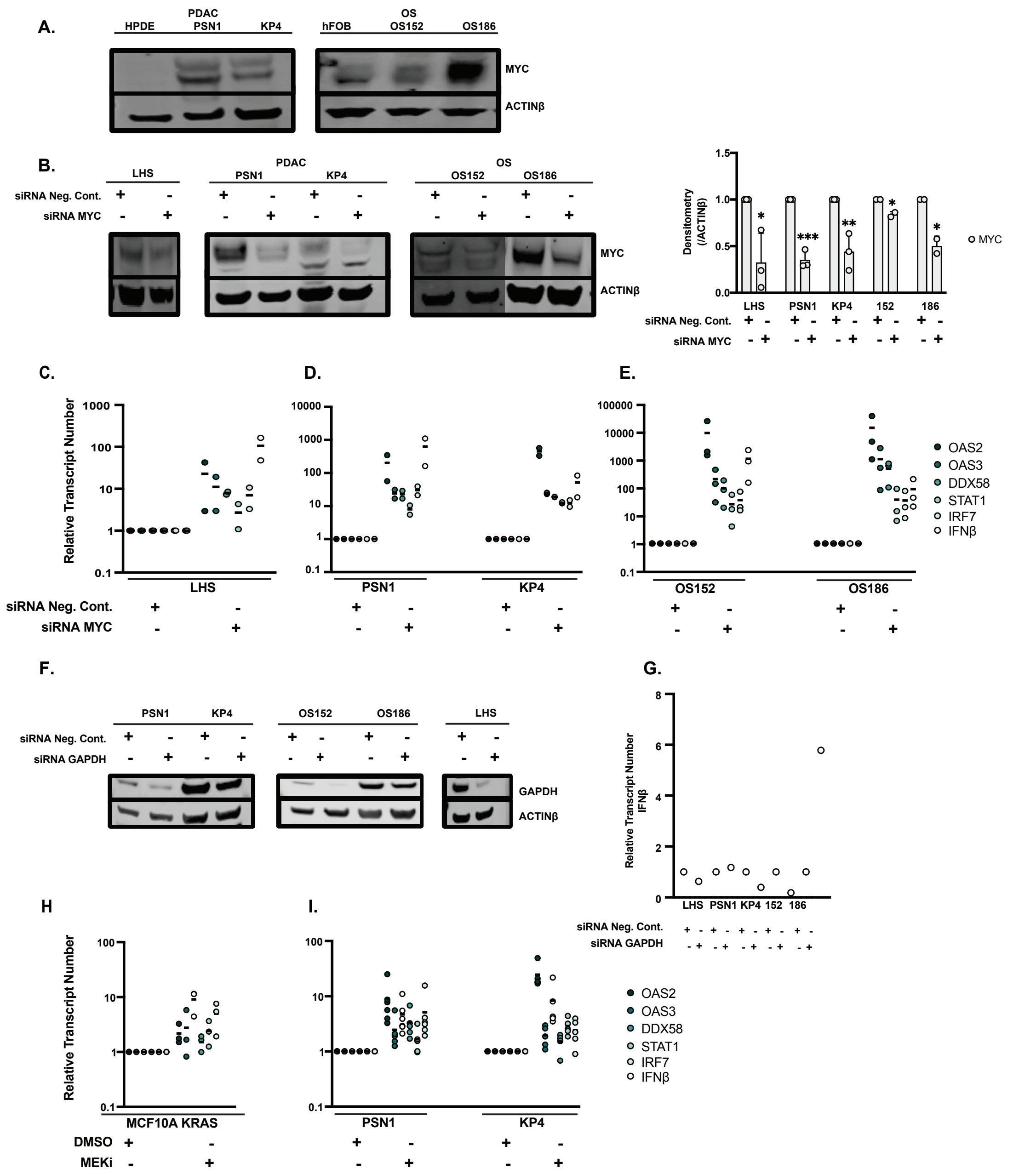

Figure S1. Knock-down and pathway inhibitors validate MYC and KRAS oncogenes suppress ISGs in cancer-derived cell lines. A. Immunoblots demonstrating increased MYC expression in PDAC cell lines and OS PDX-derived cell lines in comparison to respective HPDE and hFOB normal cells. **B.** LHS parental, PDAC, and OS cell lines were transfected with negative control siRNA or MYC siRNA. Knock-down efficiency was determined by immunoblot. Densities normalized to negative control siRNA are summarized in bar graphs. Data represent mean and standard deviation of at least two biological replicates. Statistics were calculated using Student's t-test between negative control siRNA and MYC siRNA. **C-E.** LHS parental (**C**), PDAC (**D**), and OS (**E**) cell lines were treated with negative control or MYC siRNA. Transcript levels of ISGs and IFNβ relative to GUSβ reference gene was quantified by qPCR. Data represent biological duplicates. **F-G.** As an additional control to verify that siRNA treatments do not stimulate ISG

expression, the cell types indicated were transfected with negative control siRNA or GAPDH siRNA. **F.** Knock-down efficiency was quantified by immunoblot. **G.** No dramatic change in IFNβ transcript levels were induced by GAPDH siRNA treatment. **H-I.** MCF10A KRAS (**H**) and PDAC (**I**) cell lines were treated with 2μM MEKi for 18 hours. Transcript levels of ISGs and IFNβ relative to GUSβ reference gene was quantified by qPCR. Data represent at least three biological replicates.

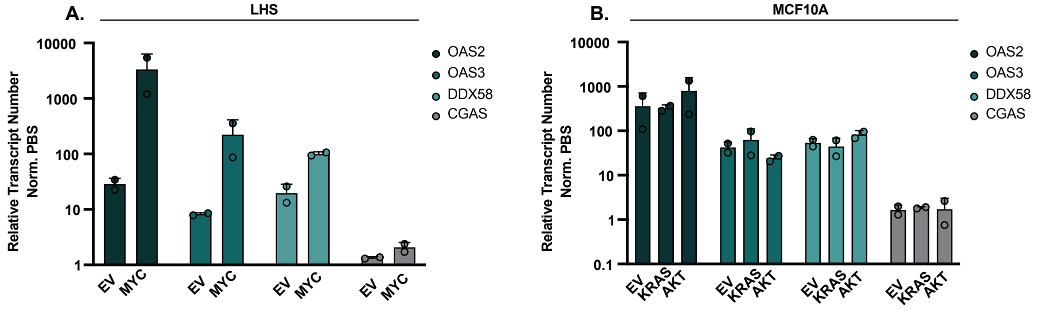

Figure S2. cGAS is not induced by IFNβ in these cell lines. A-B. LHS EV/MYC cells (**A**) and MCF10A EV/KRAS/AKT cells (**B**) were treated with 500U/mL hIFNβ or PBS for 16 hours. mRNA levels of OAS2, OAS3, DDX58, STAT1, and CGAS relative to GUSβ reference gene were quantified by qPCR. Bar graphs report mean and standard deviation of biological duplicates.

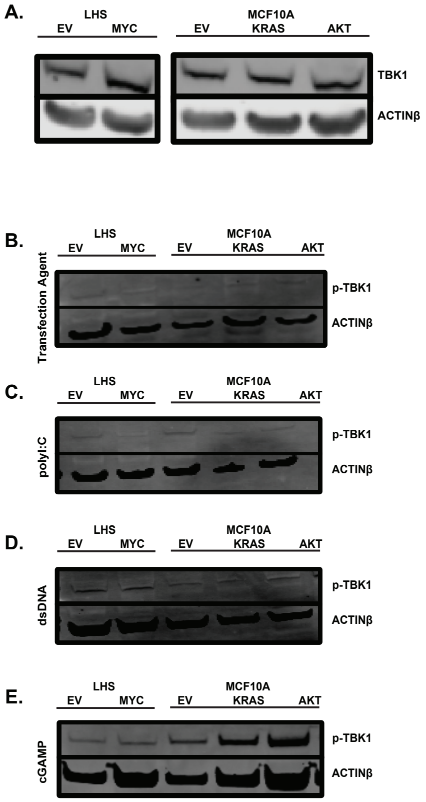

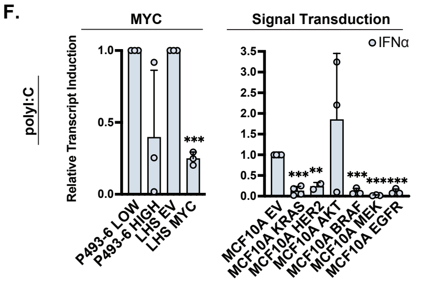

G.

RIN^e(Trans. Agent) - RIN^e(pI:C)

	R1		R2		R3	
	EV	ONC	EV	ONC	EV	ONC
MYC	2.2	0.7	4.4	0.9	NA	NA
KRAS	3.1	1.8	4.3	1.9	NA	NA
MEK	2.2	1.1	3.6	2.6	3.5	2.4
BRAF	3.6	3.2	3.5	1.4	NA	NA
HER2	3.1	0.8	4.3	1.8	NA	NA
EGFR	2.2	1.2	3.6	3.4	3.5	3.2
AKT	3.1	2.4	4.3	3.9	NA	NA

Figure S3. Cells expressing oncogenes are desensitized to dsRNA ligands. A. Baseline total TBK1 expression was immunoblotted in LHS EV/LHS MYC and MCF10A EV/MCF10A KRAS/MCF10A AKT cells. **B-E.** Cells with or without oncogenes were treated with transfection agent alone (**B**) or complexed with polyI:C (**C**), salmon dsDNA (**D**), or cGAMP (**E**). Phosphorylation at Ser172 of TBK1 was immunoblotted. **F**. Cells with or without oncogenes were treated with transfection agent alone or complexed with polyI:C. Transcript level of IFNα relative to GUSβ reference gene was quantified by qPCR. Extent of IFNα induction was calculated as the fold change in IFNα mRNA between polyI:C treatment and transfection agent alone. Data are normalized to the induction value of EV. Bar graphs represent mean and standard deviation of at least two biological replicates. Statistics were calculated using Student's t-test between EV and oncogene. **G.** Baseline-corrected RIN^e values (transfection agent treatment - polyI:C treatment) for LHS and MCF10A oncogene models. R1, R2, and R3 are biological replicates.

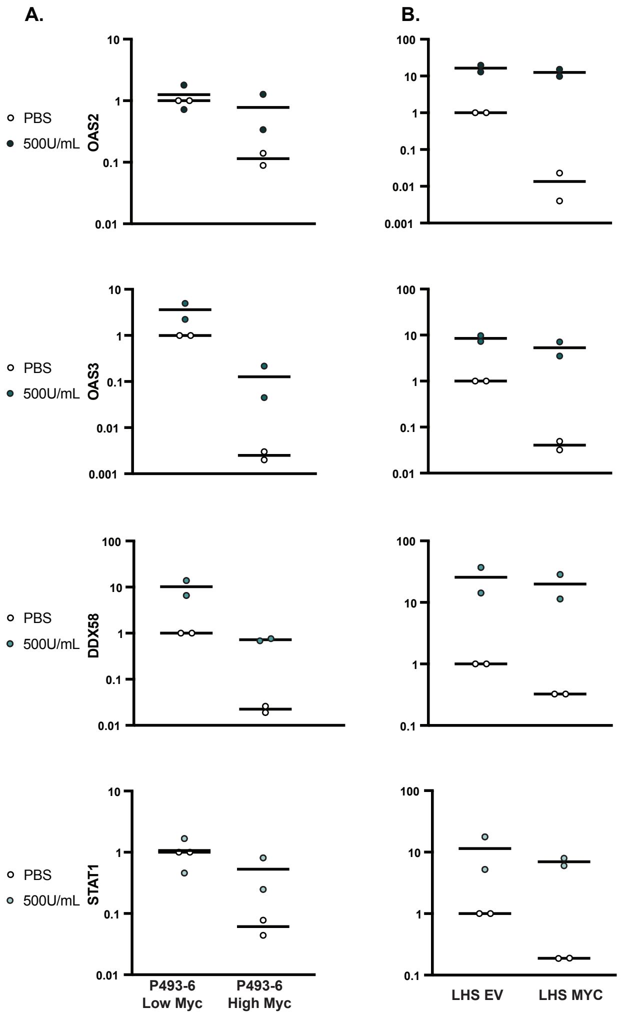

Figure S4. Cells overexpressing MYC have dramatically lower baseline expression of ISGs. A-B. Data (same as in **Figure 4C**) for P493-6 (**A**) and LHS (**B**) MYC overexpression models were re-analyzed using different normalization: transcript values were normalized to the value of non-oncogene (P493-6 Low Myc or LHS EV) treated with PBS.

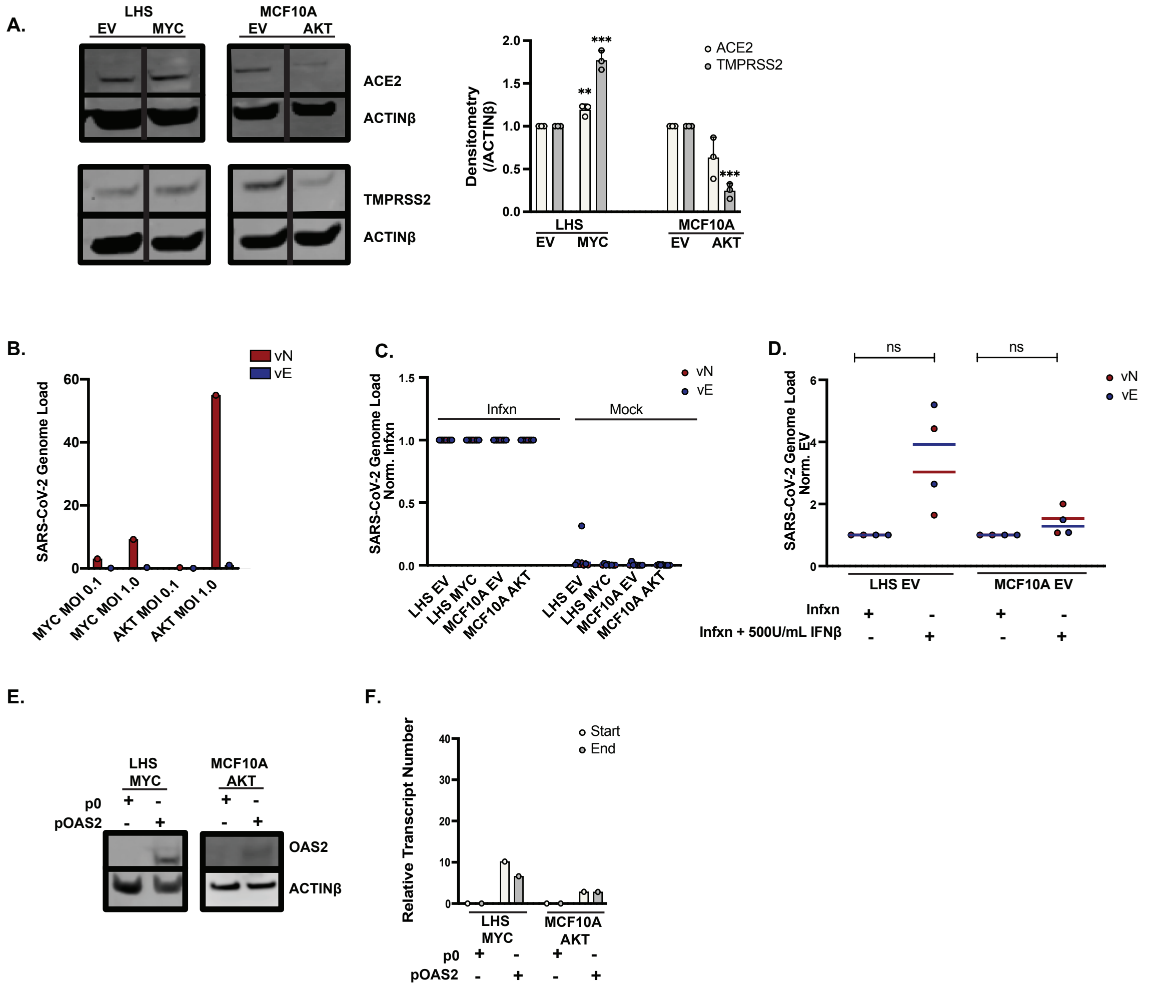

Figure S5. Controls and validations for SARS-CoV-2 assays. A. ACE2 and TMPRSS2 were immunoblotted. Densities normalized to non-oncogene cells are summarized. Bar graphs report mean and standard deviation of three biological replicates. Statistics were calculated using Student's t-test between EV and oncogene. **B.** MOIs were chosen based on experimental pre-work. LHS MYC cells had substantial viral load at MOI 0.1. In contrast, MCF10A AKT cells required 10x the amount of virus for comparable signal and were therefore treated at MOI 1.0 (viral N (vN) and E (vE) genes relative to GUSβ host reference gene). **C**. Each SARS-CoV-2 infection experiment contained mock infection controls. qPCR CT values were >37 for no infection (mock) controls. The highest CT value for an infected cell (with the lowest amount of infection) was <32. **D.** Non-oncogene LHS EV and MCF10A EV cells with or without 500U/mL IFNβ pre-treatment were infected with SARS-CoV-2. Cellular RNA was harvested and viral genome load was quantified by qPCR amplification of vN and vE genes relative to cellular GUSβ reference gene. Transcript values were normalized to untreated conditions. Data represent biological duplicates and statistics were calculated using Student's t-test between conditions. **E-F.** Cells expressing MYC and AKT oncogenes were engineered to stably overexpress OAS2 (pOAS2) or empty plasmid (p0). Cell lines were validated by western blot (**E**) and qPCR amplification of each end of the OAS2 transgene (relative to GUSβ reference gene) (**F**).

Transcript	qPCR Primers (5'-3')
IFNβ[1]	**F:** AAACTCATGAGCAGTCTGCA<br>**R:** AGGAGATCTTCAGTTTCGGAGG
pan-IFNα[1]	**F:** GTGAGGAAATACTTCCAAAGAATCAC<br>**R:** TCTCATGATTTCTGCTCTGACAA
OAS2[6]	**F:** ACCCGAACAGTTCCCCCTGGT<br>**R:** ACAAGGGTACCATCGGAGTTGCC
OAS3[6]	**F:** TGCTGCCAGCCTTTGACGCC<br>**R:** TCGCCCGCATTGCTGTAGCTG
DDX58[7]	**F:** TGTGGGCAATGTCATCAAA<br>**R:** GAAGCACTTGCTACCTCTTGC
STAT1[2]	**F:** CTAGTGGAGTGGAAGCGGAG<br>**R:** CACCACAAACGAGCTCTGAA
IRF7[3]	**F:** CCACGCTATACCATCTACCTGG<br>**R:** GCTGCTATCCAGGGAAGACACA
CGAS[8]	**F:** CGGGAGCTACTATGAGCACG<br>**R:** GCCATGTTTCTTCTTGGAAACCA
SARS-CoV-2 N[5]	**F:** CACATTGGCACCCGCAATC<br>**R:** GAGGAACGAGAAGAGGCTTG
SARS-CoV-2 E[5]	**F:** ACAGGTACGTTAATAGTTAATAGCGT<br>**R:** ATATTGCAGCAGTACGCACACA
GUSβ[4]	**F:** CTCATTTGGAATTTTGCCGATT<br>**R:** CCGAGTGAAGATCCCCTTTTTA

Table S4. Sequences of primers used in qPCR amplification(1–8).

SI References:

1. X. Yu, *et al.*, Chromatin remodeling: demethylating H3K4me3 of type I IFNs gene by Rbp2 through interacting with Piasy for transcriptional attenuation. *FASEB J.* **32**, 552–567 (2018).

2. A. Litvinchuk, *et al.*, Complement C3aR Inactivation Attenuates Tau Pathology and Reverses an Immune Network Deregulated in Tauopathy Models and Alzheimer's Disease. *Neuron* **100**, 1337-1353.e5 (2018).

3. Y. Wu, *et al.*, Function of HNRNPC in breast cancer cells by controlling the dsRNA-induced interferon response. *EMBO J.* **37** (2018).

4. J. Tratwal, B. Follin, A. Ekblond, J. Kastrup, M. Haack-Sørensen, Identification of a common reference gene pair for qPCR in human mesenchymal stromal cells from different tissue sources treated with VEGF. *BMC Mol. Biol.* **15**, 11 (2014).

5. V. M. Corman, *et al.*, Detection of 2019 novel coronavirus (2019-nCoV) by real-time RT-PCR. *Euro Surveill. Bull. Eur. Sur Mal. Transm. Eur. Commun. Dis. Bull.* **25** (2020).

6. G. M. de Freitas Almeida, *et al.*, Differential upregulation of human 2′5′ *OAS* genes on systemic sclerosis: Detection of increased basal levels of *OASL* and *OAS* 2 genes through a qPCR based assay. *Autoimmunity* **47**, 119–126 (2014).

7. P. K. Singh, S. Singh, D. Farr, A. Kumar, Interferon-stimulated gene 15 (ISG15) restricts Zika virus replication in primary human corneal epithelial cells. *Ocul. Surf.* **17**, 551–559 (2019).

8. S. Cui, *et al.*, Nuclear cGAS Functions Non-canonically to Enhance Antiviral Immunity via Recruiting Methyltransferase Prmt5. *Cell Rep.* **33**, 108490 (2020).
